# Frailty as a predictor of mortality in lung cancer survivors: evidence from a nationally representative cohort NHIS 1997–2018

**DOI:** 10.1038/s41598-025-20785-1

**Published:** 2025-11-14

**Authors:** Shu-chao Wang, Lei Peng

**Affiliations:** 1 Department of Medical Oncology, The Second Affiliated Hospital of Shandong First Medical University, Taian, 271000 Shangdong Province China; 2 Faculty of Medicine, University of Heidelberg, 69115 Heidelberg, Germany

**Keywords:** Frailty, Lung cancer survivors, Mortality, Survival analysis, FRAIL scale, Lung cancer, Epidemiology, Disease-free survival

## Abstract

Frailty is a syndrome characterized by decreased physiological reserve and increased vulnerability to adverse health outcomes. Its impact on lung cancer survivors remains inadequately explored. This study examined the association between frailty and all-cause mortality in a nationally representative cohort. We analyzed data from the National Health Interview Survey (NHIS) (1997–2018) linked to the National Death Index through 2019. Frailty was assessed using a modified FRAIL scale, categorizing participants as robust, pre-frail, or frail. Cox proportional hazards models were used to evaluate the association between frailty and all-cause mortality, adjusting for demographic, socioeconomic, and health-related factors. Among 1,778 lung cancer survivors, 63.7% were robust, 13.1% were pre-frail, and 23.2% were frail. Frailty prevalence was significantly higher in lung cancer survivors than in cancer-free participants (*P* < 0.001). Kaplan–Meier analysis demonstrated significantly reduced survival probability with increasing frailty (*log-rank P* < 0.001). In fully adjusted Cox models, frailty was associated with a 2.12-fold increased risk of mortality (95% CI, 1.68–2.50, *P* < 0.001), and pre-frailty was also associated with increased risk (*HR* = 1.55; 95% CI, 1.27–1.90, *P* < 0.001). The impact of frailty on mortality was more pronounced in younger survivors and men. Frailty is a significant predictor of all-cause mortality in lung cancer survivors. Routine frailty assessments and targeted interventions may help improve long-term outcomes in this population.

## Introduction

Lung cancer remains one of the leading causes of cancer-related mortality worldwide, with significant advancements in early detection and treatment extending survival for many patients^1–3^. However, prolonged survival in lung cancer survivors does not necessarily equate to preserved functional status and quality of life^4–6^. Frailty, a multidimensional syndrome characterized by decreased physiological reserve and increased vulnerability to adverse health outcomes, has emerged as a critical determinant of long-term prognosis in aging populations^7–10^. While frailty has been extensively studied in geriatric medicine^11–13^, its implications for cancer survivorship, particularly among individuals with lung cancer, remain inadequately explored^7,14^.

Previous investigations in mixed oncology cohorts have demonstrated that frailty predicts adverse outcomes such as treatment complications, functional decline, hospitalization, and mortality^15,16^. Evidence on frailty specifically in lung cancer survivors remains limited and has largely focused on surgical populations. In a single-institution series of 439 patients undergoing pulmonary resection for non–small cell lung cancer, the 11-item modified frailty index independently predicted 90-day unplanned readmissions, postoperative complications, and poorer overall survival^17^. In a much larger cohort of 36,587 lung cancer resections captured in the Society of Thoracic Surgeons database, the 5-item modified frailty index (mFI-5) demonstrated independent, incremental associations with virtually all postoperative complications and administrative outcomes, including 30-day mortality, with predictive performance comparable to, or exceeding, traditional risk tools^18^. More broadly, a recent systematic review and meta-analysis of frailty in lung cancer patients confirmed that frailty is significantly associated with worse overall survival across studies^19^. However, these investigations are constrained by their surgical focus, varied frailty definitions, and relatively short postoperative follow-up, leaving the long-term prognostic implications of frailty among the wider population of lung cancer survivors, many of whom do not undergo surgical resection, largely undefined. Despite these insights, studies to date have been limited by small, single-center samples, heterogeneous frailty measures, and a lack of large-scale, population-based evidence specifically focused on the long-term prognostic implications of frailty among lung cancer survivors^20–24^.

To address these gaps, we applied the validated FRAIL scale^25,26^ to classify lung cancer survivors as robust, pre-frail, or frail, and then quantified frailty prevalence in a large, representative cohort. We evaluated the independent association between frailty status and all-cause mortality, adjusting for demographic and clinical covariates, and examined whether these associations differed by age group (< 60 *vs.* ≥ 60 years) and by sex. We hypothesized that frailty would emerge as a strong, independent predictor of mortality across subgroups, thereby informing the stratification of survivorship care.

By leveraging a comprehensive cohort with extended follow-up, our study aims to provide generalizable evidence on the prognostic significance of frailty in lung cancer survivorship, underscoring the need for routine frailty assessment and targeted interventions to improve long-term outcomes in this vulnerable population.

## Methods

### Study design and population selection

This study utilized data from the NHIS spanning the years 1997 to 2018. The NHIS is a nationally representative, cross-sectional survey conducted by the National Center for Health Statistics (NCHS) to assess health status, behaviors, and sociodemographic factors among the U.S. population.

A total of 671,696 participants were initially identified from the NHIS 1997–2018 dataset. Sequential exclusion criteria were applied to ensure data completeness and study relevance. Participants with missing data on frailty assessment (N = 28,461) were excluded, followed by those with missing information on key covariates (N = 47,238). Individuals with a history of cancer other than lung cancer (N = 467) were also excluded. Additionally, participants with missing mortality outcome data (N = 5742) were removed.

**Fig. 1:**
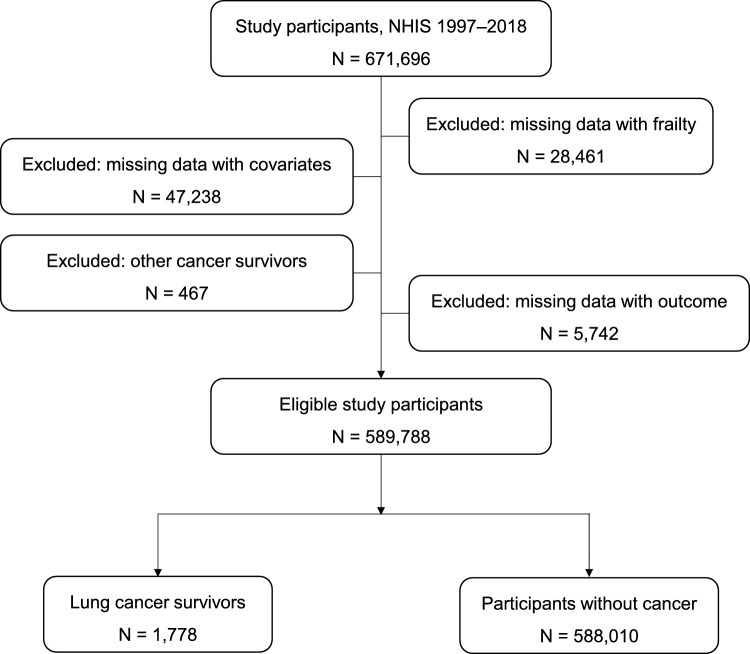
Flow chart of study participants selection.

After these exclusions, 589,788 individuals remained eligible for analysis. Among them, 1,778 were identified as lung cancer survivors, while 588,010 had no history of cancer. The final analytic cohort consisted of these two groups, allowing for the examination of frailty status in relation to all-cause mortality (Fig. 1).

### Frailty assessment using the FRAIL scale

Frailty was assessed using a modified FRAIL scale^25,27^, a validated screening instrument originally developed by the Geriatric Advisory Panel of the International Society for Nutrition and Aging. For this study, we derived frailty measures from the 1997–2018 NHIS, utilizing self-reported questionnaire responses. The FRAIL scale consists of five domains: Fatigue, Resistance, Ambulation, Illness, and Low body mass index (BMI)^28^.

Fatigue was assessed using NHIS survey questions regarding the frequency of experiencing tiredness or low energy over a predefined recall period. Responses indicating frequent or persistent fatigue were scored as 1, whereas those reflecting minimal or no fatigue were scored as 0.

Resistance was evaluated based on self-reported difficulty in ascending or descending 12 steps without assistance or assistive devices. Ambulation was assessed by asking whether the participant experienced difficulty walking 100 yards on level ground (approximately the length of a football field or a city block) unaided. Participants reporting no difficulty in either task were assigned a score of 0, while those reporting any degree of difficulty received a score of 1.

The illness component was derived from self-reported physician-diagnosed chronic conditions. Participants reporting five or more conditions from a predefined list of 12 diseases—angina, anxiety disorder, arthritis, asthma, chronic obstructive pulmonary disease, coronary heart disease, dementia, diabetes, myocardial infarction, hyperlipidemia, hypertension, and stroke—were assigned a score of 1, while those reporting fewer than five were assigned a score of 0.

Low BMI was defined as a body mass index (BMI) below 18.5 kg/m^2^, with affected individuals receiving a score of 1 and all others assigned a score of 0.

The total FRAIL scale score ranged from 0 to 5, with participants categorized as frail (scores of 3–5), pre-frail (scores of 1–2), or robust (score of 0)^25^.

### Covariates

In this study, we utilized data from the NHIS to assess various covariates, including age, sex, race/ethnicity, education level, health insurance status, marital status, geographic region, and depression status, time since cancer diagnosis, number of cancer diagnoses, and number of comorbidities. The NHIS is a nationally representative, cross-sectional survey conducted annually by the NCHS to monitor the health of the civilian non-institutionalized U.S. population.

Participants reported their age in years at the time of the interview and self-identified their sex as either male or female. These demographic variables are standard components of the NHIS and are collected through direct questioning during household interviews. ace and Hispanic origin were determined based on self-identification. Participants were asked to select one or more races that they considered themselves to be, and to indicate whether they were of Hispanic or Latino origin. For analytical purposes, race/ethnicity was categorized into White, Black, Asian, and Other. Educational attainment was assessed by inquiring about the highest level of school completed or the highest degree received. Responses were categorized as less than high school, high school graduate, and more than high school. Participants were asked about their health insurance coverage at the time of the interview, including private health insurance, Medicare, Medicaid, or other government-sponsored health plans. Responses were dichotomized into insured (yes) or uninsured (no). Marital status was determined by asking participants to describe their current marital situation, with responses categorized as married or unmarried. The NHIS classifies participants’ residence into four geographic regions: Northeast, Midwest, South, and West, based on the U.S. Census Bureau’s regional definitions. Depression was assessed through self-reported data, where participants indicated whether they had ever been told by a healthcare professional that they had depression. This method of assessment aligns with approaches used in prior studies analyzing NHIS data. By employing these standardized NHIS measures, we ensured the reliability and validity of the covariate data utilized in our analyses.

Information on cancer history was also based on self-reported responses in the NHIS. Participants who responded “yes” to the question “Have you ever been told by a doctor or other health professional that you had cancer or a malignancy of any kind?” were classified as having a history of cancer. Time since cancer diagnosis was calculated as the difference between the year of interview and the self-reported year of the first cancer diagnosis, and was categorized as < 2 years or ≥ 2 years. The number of cancer diagnoses was derived from the total number of different cancer types reported by each participant, and was classified as 1 or ≥ 2. Both variables were included as categorical covariates in multivariable analyses to account for the potential confounding effect of cancer history. The number of comorbidities was defined as the total count of self-reported physician-diagnosed chronic conditions (angina, anxiety disorder, arthritis, asthma, chronic obstructive pulmonary disease, coronary heart disease, dementia, diabetes, myocardial infarction, hyperlipidemia, hypertension, and stroke) and categorized for analysis as 0–1, 2–3, or ≥ 4.

### All-cause mortality

To facilitate mortality follow-up, the NHIS data from 1997 to 2018 were linked to mortality outcomes through the NDI, enabling comprehensive mortality follow-up through December 31, 2019. This linkage was facilitated by the NCHS Data Linkage Program, which employs a probabilistic matching methodology to align NHIS records with NDI death certificate data. This process allows for the assessment of vital status and cause-specific mortality among survey participants.

The linkage process adhered to stringent confidentiality protocols to protect respondent privacy. In the public-use Linked Mortality Files (LMF), data perturbation techniques were applied to reduce the risk of participant re-identification. For select records, synthetic data were substituted for follow-up time or underlying cause of death; however, information regarding vital status remained unaltered.

Utilizing the NHIS Linked Mortality Files, we conducted a comprehensive analysis of mortality outcomes among study participants. This approach allowed us to investigate the associations between frailty status and all-cause mortality, leveraging the rich health and demographic data collected by the NHIS.

### Statistical analysis

All statistical analyses were conducted using SAS software, version 9.4 (Cary, North Carolina, USA) and R software (version 4.3.1; https://www.R-project.org). Differences in categorical variables between groups were assessed using the chi-square (*χ*^*2*^) test, while differences in continuous variables were evaluated using one-way analysis of variance (ANOVA).

Survival analyses were conducted using Kaplan–Meier curves to estimate survival probabilities, with comparisons between groups assessed using the log-rank test. Multivariable Cox proportional hazards regression models were employed to evaluate the association between frailty status and all-cause mortality, adjusting for potential confounders. The proportional hazards assumption was assessed using Schoenfeld residuals.

All statistical tests were two-sided, and a *P*-value of less than 0.05 was considered to indicate statistical significance.

## Results

### Study population characteristics

Table 1 presents the baseline characteristics of lung cancer patients stratified by frailty status. Among the 1,778 individuals included in the study, 63.7% were classified as robust, 13.1% as pre-frail, and 23.2% as frail. The mean age varied significantly across frailty groups (*P* = 0.003), with frail individuals being the oldest (mean ± SD: 70.6 ± 9.5 years) compared to those classified as robust (67.7 ± 12.1 years) and pre-frail (68.3 ± 10.8 years). The distribution of sex did not differ significantly across frailty categories (*P* = 0.146), though women constituted a slightly higher proportion in the robust group (51.7%) compared to the pre-frail (45.5%) and frail (48.1%) groups.

**Table 1:** Baseline characteristics of lung cancer patients according to frailty status, NHIS 1997–2018.

Characteristics	Robust 1133 (63.7%)	Pre-frail 233 (13.1%)	Frail 412 (23.2%)	*P*-value ^a^
Age, years	67.7 (12.1)	68.3 (10.8)	70.6 (9.5)	0.003
Sex, %				0.146
Women	586 (51.7)	106 (45.5)	198 (48.1)	
Men	547 (48.3)	127 (54.5)	214 (51.9)	
Race/ethnicity, %				< 0.001
White	698 (61.6)	154 (66.1)	79 (19.2)	
Black	105 (9.3)	28 (12.0)	16 (3.9)	
Asian	8 (0.7)	1 (0.4)	0 (0.0)	
Other	322 (28.4)	50 (21.5)	317 (76.9)	
Education level, %				0.014
< High school	523 (46.2)	116 (49.8)	227 (55.1)	
High school graduate	221 (19.5)	40 (17.3)	67 (16.2)	
> High school	389 (34.3)	77 (32.9)	118 (28.7)	
Health insurance, %				0.007
Yes	1118 (98.7)	227 (97.4)	396 (96.1)	
No	15 (1.3)	6 (2.6)	16 (3.9)	
Marital status, %				0.824
Married	477 (42.1)	93 (39.9)	171 (41.5)	
Unmarried	656 (57.9)	140 (60.1)	241 (58.5)	
Region, %				0.911
Northeast	210 (19.1)	37 (16.1)	65 (17.5)	
Midwest	260 (23.6)	62 (27.0)	93 (24.9)	
South	436 (39.6)	90 (39.1)	149 (39.9)	
West	194 (17.6)	41 (17.8)	66 (17.7)	
Depression, %				< 0.001
No	1093 (96.5)	212 (91.0)	381 (92.5)	
Yes	40 (3.5)	21 (9.0)	31 (7.5)	
Time since cancer diagnosis				< 0.001
< 2 years	140 (12.4%)	42 (18.1%)	108 (26.3%)	
≥ 2 years	993 (87.6%)	191 (81.9%)	304 (73.7%)	
Number of cancer diagnoses				< 0.001
1	992 (87.5%)	197 (84.7%)	321 (78.0%)	
≥ 2	141 (12.5%)	36 (15.3%)	91 (22.0%)	
Number of comorbidities				< 0.001
0–1	700 (61.8%)	120 (51.5%)	100 (24.3%)	
2–3	300 (26.5%)	80 (34.3%)	180 (43.7%)	
≥ 4	133 (11.7%)	33 (14.2%)	132 (32.0%)	

Values are means (SDs) for continuous variables and percentages for categorical variables.

^**a**^ Group differences were assessed using analysis of variance (ANOVA) for continuous variables and the chi-square test for categorical variables.

Significant disparities were observed in racial/ethnic composition across frailty groups (*P* < 0.001). Among robust participants, the majority were White (61.6%), followed by those identifying as Other (28.4%), Black (9.3%), and Asian (0.7%). In contrast, the frail group had a markedly higher proportion of individuals classified as Other (76.9%), while the proportion of White participants was substantially lower (19.2%). The percentage of Black participants was also lower in the frail group (3.9%) compared to the robust and pre-frail groups. Education level was significantly associated with frailty status (*P* = 0.014). A higher proportion of frail participants had less than a high school education (55.1%) compared to those who were robust (46.2%) or pre-frail (49.8%). Conversely, the proportion of individuals with more than a high school education was highest in the robust group (34.3%) and lowest in the frail group (28.7%). The prevalence of health insurance coverage was high across all groups but showed a significant difference (*P* = 0.007), with insurance coverage being slightly lower among frail participants (96.1%) compared to robust (98.7%) and pre-frail (97.4%) individuals. Marital status did not differ significantly by frailty category (*P* = 0.824), with similar proportions of married and unmarried individuals across the groups. There were no significant differences in geographic distribution among frailty groups (*P* = 0.911). The largest proportion of participants resided in the South (~ 39% across all frailty categories), followed by the Midwest (23.6–27.0%), the Northeast (16.1–19.1%), and the West (17.6–17.8%). The prevalence of self-reported depression differed significantly among frailty groups (*P* < 0.001). Depression was least common in the robust group (3.5%), while the prevalence was higher among pre-frail (9.0%) and frail (7.5%) individuals.

Significant differences were also observed across frailty groups for time since cancer diagnosis (*P* < 0.001). The proportion of survivors diagnosed less than 2 years before the survey increased from 12.4% in the robust group to 18.1% in the pre-frail group and 26.3% in the frail group, whereas those with ≥ 2 years since diagnosis comprised 87.6% of robust, 81.9% of pre-frail, and only 73.7% of frail survivors. Similarly, the number of cancer diagnoses differed significantly (*P* < 0.001): a single diagnosis was reported by 87.5% of robust, 84.7% of pre-frail, and 78.0% of frail participants, while multiple diagnoses (≥ 2) were more common among frail survivors (22.0%) than pre-frail (15.3%) or robust (12.5%) groups. Finally, comorbidity burden showed the most pronounced gradient (*P* < 0.001): 0–1 comorbidities were present in 61.8% of robust, 51.5% of pre-frail, and only 24.3% of frail survivors; the 2–3 comorbidities category rose from 26.5% in robust to 34.3% in pre-frail and 43.7% in frail groups; and ≥ 4 comorbidities were reported by 11.7% of robust, 14.2% of pre-frail, and 32.0% of frail individuals.

In summary, frail survivors differed markedly from their robust counterparts on almost every dimension: they were older, more often had less than a high-school education, and were slightly less likely to be insured. Frail individuals also exhibited a higher burden of depression and were more frequently diagnosed within the past two years and with multiple cancer primaries. Moreover, comorbidity counts rose steeply across frailty categories—with frail patients most likely to report ≥ 4 chronic conditions. Finally, the racial/ethnic makeup varied substantially by frailty status: those classified as frail were disproportionately represented in the “Other” category and far less likely to be White or Black than robust survivors.

### Comparison of frailty status between lung cancer survivors and healthy participants

**Fig. 2:**
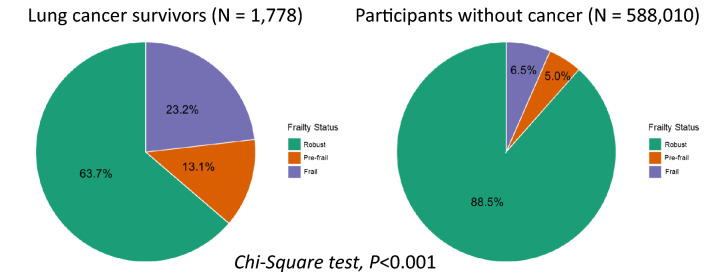
Distribution of frailty status among lung cancer survivors and cancer-free participants. This figure presents the distribution of frailty status among lung cancer survivors (N = 1778) and participants without a history of cancer (N = 588,010) from the National Health Interview Survey (NHIS) 1997–2018. Frailty was categorized using the modified FRAIL scale as robust (score = 0), pre-frail (score = 1–2), and frail (score = 3–5). In lung cancer survivors, 63.7% were classified as robust, 13.1% as pre-frail, and 23.2% as frail. Among cancer-free participants, the prevalence of frailty was significantly lower, with 88.5% categorized as robust, 5.0% as pre-frail, and 6.5% as frail. A chi-square test demonstrated a statistically significant difference in frailty distribution between lung cancer survivors and cancer-free participants (*P* < 0.001).

Frailty status distribution differed significantly between lung cancer survivors and participants without a history of cancer (*P* < 0.001, Fig. 2). Among lung cancer survivors, 63.7% were classified as robust, 13.1% as pre-frail, and 23.2% as frail. In contrast, the prevalence of frailty was substantially lower among participants without cancer, with 88.5% classified as robust, 5.0% as pre-frail, and only 6.5% as frail.

The proportion of frail individuals was more than threefold higher among lung cancer survivors compared to participants without cancer (23.2% *vs.* 6.5%), while the percentage of robust individuals was significantly lower in the lung cancer group (63.7% *vs.* 88.5%). Similarly, the prevalence of pre-frailty was more than twice as high in lung cancer survivors as in cancer-free participants (13.1% *vs.* 5.0%).

### Survival probability across different frailty statuses in lung cancer survivors

**Fig. 3:**
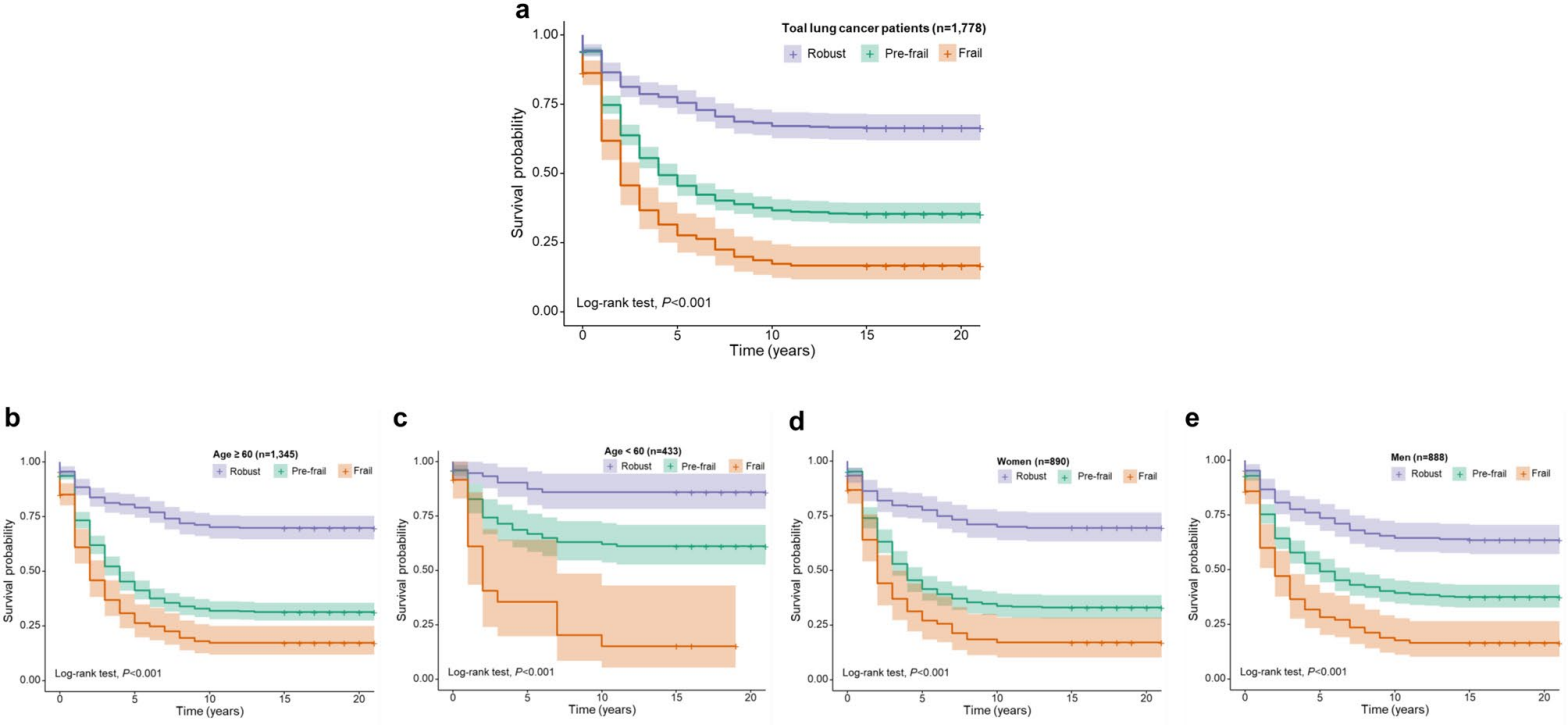
Survival probability by frailty status among lung cancer survivors. Kaplan–Meier survival curves illustrate differences in survival probabilities across frailty categories (robust, pre-frail, and frail) among lung cancer survivors in NHIS 1997–2018. (**a**) Overall lung cancer survivors show a significant decline in survival with increasing frailty (*log-rank P* < 0.001). (**b**) Among survivors aged ≥ 60 years, survival curves for pre-frail and frail groups are closely aligned, indicating comparable mortality risks. (**c**) In survivors aged < 60 years, frailty remains strongly associated with mortality, but limited sample size results in wide confidence intervals. (**d**) Among women, pre-frail and frail individuals have similar survival patterns, resembling the older age group. (**e**) Among men, survival curves mirror those of the overall cohort, with frailty significantly impacting survival (*log-rank P* < 0.001).

Kaplan–Meier survival analysis demonstrated significant differences in survival probabilities across frailty categories among lung cancer survivors (*log-rank P* < 0.001, Fig. 3a). Individuals classified as frail exhibited the lowest survival probability, followed by those categorized as pre-frail, while robust participants had the highest survival probability throughout the follow-up period.

Stratification by age revealed persistent associations between frailty status and survival, though patterns varied between older and younger participants. Among lung cancer survivors aged ≥ 60 years, frailty remained significantly associated with reduced survival (*log-rank P* < 0.001, Fig. 3b). However, survival curves for the pre-frail and frail groups closely overlapped, suggesting a potentially comparable mortality risk in these two groups. In contrast, among lung cancer survivors aged < 60 years, the distinction between survival curves for robust, pre-frail, and frail individuals remained significant (*log-rank P* < 0.001, Fig. 3c). However, limited sample size in this subgroup resulted in wide confidence intervals, indicating greater uncertainty in risk estimation.

Sex-stratified analyses further supported the association between frailty and mortality risk. Among women, survival probabilities differed significantly by frailty status (*log-rank P* < 0.001, Fig. 3d), but pre-frail and frail individuals exhibited similar survival curves, resembling the pattern observed in older lung cancer survivors (≥ 60 years). Conversely, among men, survival probability varied significantly across frailty groups (*log-rank P* < 0.001, Fig. 3e), and the survival curves closely resembled those observed in the overall lung cancer cohort.

### Longitudinal association between frailty and all-cause mortality

The association between frailty status and all-cause mortality among lung cancer survivors is presented in Table 2. In both the age- and sex-adjusted model and the fully adjusted multivariate model, frailty status was significantly associated with an increased risk of mortality, demonstrating a clear dose–response relationship.

**Table 2:** Association between frailty status and all-cause mortality among lung cancer patients, NHIS 1997–2018.

Frailty status	Age- and sex-adjusted model^a^			Multivariate adjusted model^b^		
	HR	95%CI	*P*-value	HR	95%CI	*P*-value
Total lung cancer patients						
Robust	Ref	Ref		Ref	Ref	
Pre-frail	1.68	1.38–2.04	< 0.001	1.55	1.27–1.90	< 0.001
Frail	2.31	1.86–2.70	< 0.001	2.12	1.68–2.50	< 0.001
≥ 60 years old						
Robust	Ref	Ref		Ref	Ref	
Pre-frail	1.59	1.30–1.96	< 0.001	1.48	1.20–1.83	< 0.001
Frail	2.13	1.85–2.42	< 0.001	1.98	1.75–2.25	< 0.001
< 60 years old						
Robust	Ref	Ref		Ref	Ref	
Pre-frail	3.16	1.83–5.46	< 0.001	1.78	1.01–3.17	0.048
Frail	4.01	3.75–4.33	< 0.001	2.95	2.10–3.94	< 0.001
Women						
Robust	Ref	Ref		Ref	Ref	
Pre-frail	1.55	1.16–2.05	0.003	1.35	1.02–1.80	0.030
Frail	2.84	2.38–3.35	< 0.001	2.30	1.85–2.86	< 0.001
Men						
Robust	Ref	Ref		Ref	Ref	
Pre-frail	1.82	1.39–2.38	< 0.001	1.70	1.28–2.25	< 0.001
Frail	3.24	2.59–3.93	< 0.001	2.88	2.20–3.70	< 0.001

^**a**^ Cox regression model adjusted for age and sex.

^**b**^ Cox regression model adjusted for age, sex, race/ethnicity, education level, health insurance, marital status, region, and depression, time since cancer diagnosis, number of cancer diagnoses, and number of comorbidities.

*HR* hazards ratio, *CI* confidence interval.

Among all lung cancer survivors, individuals classified as pre-frail had a significantly higher risk of all-cause mortality compared to their robust counterparts (*HR* = 1.55, 95% *CI* 1.27–1.90, *P* < 0.001). The risk was even more pronounced among frail participants, with a more than twofold increased hazard of mortality (*HR* = 2.12, 95% *CI* 1.68–2.50, *P* < 0.001).

In the subgroup of lung cancer survivors aged ≥ 60 years, frailty remained a strong predictor of mortality. Compared with the robust group, pre-frail individuals had a 48% higher mortality risk (*HR* = 1.48, 95% *CI* 1.20–1.83, *P* < 0.001), while frail individuals had more than twice the risk of death (*HR* = 1.98, 95% *CI* 1.75–2.25, *P* < 0.001). In contrast, among lung cancer survivors aged < 60 years, frailty had an even greater impact on survival. Pre-frail individuals exhibited a 1.78-fold increased risk of mortality (*HR* = 1.78, 95% *CI* 1.01–3.17, *P* = 0.048), while frail individuals had more than three times the risk of death compared to robust individuals (*HR* = 2.95, 95% *CI* 2.10–3.94, *P* < 0.001). Notably, the confidence intervals for this subgroup were wider, likely reflecting the smaller sample size.

The association between frailty and mortality remained significant in both women and men. Among women, pre-frail individuals had a 35% increased mortality risk (*HR* = 1.35, 95% *CI* 1.02–1.80, *P* = 0.030), while frail women had a 2.41-fold increased risk of mortality (*HR* = 2.30, 95% *CI* 1.85–2.86, *P* < 0.001). Among men, the impact of frailty on mortality was even more pronounced. Pre-frail men had a 70% increased risk of death compared to their robust counterparts (*HR* = 1.70, 95% *CI* 1.28–2.25, *P* < 0.001), while frail men had a more than threefold higher risk of mortality (*HR* = 2.88, 95% *CI* 2.20–3.70, *P* < 0.001). The effect estimates in men closely resembled those observed in the overall lung cancer cohort.

## Discussion

In this nationally representative cohort of lung cancer survivors, we found that frailty was significantly associated with all-cause mortality, demonstrating a clear dose–response relationship. Frail individuals exhibited the highest mortality risk, followed by pre-frail individuals, while robust survivors had the most favorable prognosis. These associations remained robust after adjusting for key demographic and clinical covariates, highlighting frailty as an independent predictor of mortality in lung cancer survivors.

Our findings align with previous studies demonstrating the detrimental impact of frailty on survival in cancer populations. Prior research has established frailty as a key determinant of adverse outcomes, including hospitalization, functional decline, and mortality, in older adults and individuals undergoing cancer treatment^29,30^. However, most studies have focused on specific clinical settings or smaller cohorts, limiting their generalizability^31–33^. The present study extends this evidence by utilizing a large, population-based dataset with long-term follow-up, providing robust epidemiologic data on frailty and survival among lung cancer survivors.

Furthermore, our study contributes novel insights by comparing frailty prevalence between lung cancer survivors and cancer-free individuals. The substantially higher prevalence of frailty in lung cancer survivors underscores the profound physiological burden of cancer and its treatment ^19,34–36^. This observation reinforces the need for routine frailty assessments in oncology practice, as early identification of vulnerable individuals may facilitate targeted interventions to improve long-term outcomes.

Stratified analyses revealed important age- and sex-related differences in the frailty-mortality relationship. Among lung cancer survivors aged ≥ 60 years, frailty was strongly associated with increased mortality, but the survival curves for pre-frail and frail individuals overlapped, suggesting that even mild impairments in physiological resilience confer substantial mortality risk in older adults^37,38^. In contrast, among survivors aged < 60 years, frailty had a more pronounced impact on survival, with frail individuals exhibiting more than a threefold increased risk of mortality. However, the smaller sample size in this subgroup resulted in wider confidence intervals, highlighting the need for further research in younger lung cancer survivors.

Sex-stratified analyses also revealed notable differences. While frailty was a strong predictor of mortality in both men and women, pre-frail and frail women had more similar survival trajectories, mirroring the pattern observed in older lung cancer survivors. This suggests that sex-related biological or behavioral factors may modulate the impact of frailty on survival^20,39^, warranting further investigation. Conversely, among men, frailty had a more distinct and pronounced effect on mortality, with risk estimates closely resembling those observed in the overall lung cancer cohort.

These findings have significant implications for both clinical practice and public health. First, our study highlights the critical role of frailty assessment in lung cancer survivorship care. Given its strong association with mortality, frailty screening should be integrated into routine oncology practice to identify high-risk individuals who may benefit from early intervention. While oncologic decision-making has traditionally been guided by chronological age, our results underscore the importance of biological frailty as a more precise indicator of vulnerability.

Second, targeted interventions to mitigate frailty-related risks are urgently needed. Multidisciplinary approaches, including exercise programs, nutritional support, and comprehensive geriatric assessments, have shown promise in improving outcomes among frail individuals. Tailored survivorship care plans that incorporate frailty assessment may optimize long-term health in lung cancer survivors, particularly among those at the highest risk.

A key strength of this study is the use of a nationally representative, population-based cohort with long-term mortality follow-up, allowing for robust, generalizable estimates of the frailty-mortality association. Additionally, we employed a validated frailty measure (the FRAIL scale) and adjusted for a comprehensive set of covariates to minimize confounding.

However, several limitations should be acknowledged. First, frailty was assessed using self-reported data, which may be subject to recall bias. Second, although we adjusted for a wide range of confounders, residual confounding from unmeasured factors such as cancer stage, treatment modalities, and comorbidities remains possible. Third, the sample size for younger lung cancer survivors was relatively small, leading to wider confidence intervals and limiting the precision of estimates in this subgroup. Future research with larger cohorts is needed to validate our findings and explore mechanisms underlying the frailty-mortality relationship in lung cancer survivors.

In conclusion, frailty is a strong and independent predictor of all-cause mortality in lung cancer survivors, with significant variations by age and sex. The disproportionately high burden of frailty among lung cancer survivors compared to cancer-free individuals underscores its clinical relevance in oncology care. Our findings highlight the urgent need for routine frailty assessments and targeted interventions to improve outcomes in this vulnerable population. Future studies should further explore the underlying biological and behavioral mechanisms linking frailty to mortality, with the goal of developing precision strategies for frailty management in lung cancer survivors.

## Data Availability

Publicly available datasets were analyzed in this study. This data can be found here: http://www.cdc.gov/nchs/nhis.htm.
